# UHPLC-ESI-Orbitrap-MS/MS Untargeted Metabolomic Profiling and In Vitro Antioxidant and Antibacterial Activity of Different *Achillea clypeolata* Sibth. & Sm. Extracts

**DOI:** 10.3390/foods15081367

**Published:** 2026-04-15

**Authors:** Mariya Choneva, Ivica Dimov, Daniela Mollova, Merlin Esad, Plamen Stoyanov, Rumen Mladenov, Tsvetelina Mladenova, Krasimir Todorov, Anelia Bivolarska

**Affiliations:** 1 Department of Medical Biochemistry, Faculty of Pharmacy, Medical University of Plovdiv, 15A Vasil Aprilov Blvd., 4002 Plovdiv, Bulgaria; ivica.dimov@mu-plovdiv.bg (I.D.); merlin.esad@mu-plovdiv.bg (M.E.); anelia.bivolarska@mu-plovdiv.bg (A.B.); 2 Department of Biochemistry and Microbiology, Faculty of Biology, University of Plovdiv “Paisii Hilendarski”, 4000 Plovdiv, Bulgaria; dmollova.bio@gmail.com; 3 Department of Bioorganic Chemistry, Faculty of Pharmacy, Medical University of Plovdiv, 15A Vasil Aprilov Blvd., 4002 Plovdiv, Bulgaria; pstoyanov@uni-plovdiv.bg (P.S.); rummlad@uni-plovdiv.bg (R.M.); cmladenova@uni-plovdiv.bg (T.M.); ktodorov@uni-plovdiv.bg (K.T.); 4 Department of Botany and Biological Education, Faculty of Biology, University of Plovdiv “Paisii Hilendarski”, 24 Tsar Assen Str., 4000 Plovdiv, Bulgaria

**Keywords:** UHPLC-ESI-Orbitrap-MS/MS, *Achillea clypeolata*, metabolomic profiling, polyphenols, flavonoids, antioxidant activity, antibacterial activity

## Abstract

*Achillea clypeolata* Sibth. & Sm. is an endemic Balkan species traditionally used in Bulgarian folk medicine. The present study aimed to conduct untargeted metabolomic profiling and characterization of 70% methanol, 70% ethanol, and water *A. clypeolata* extracts via the UHPLC-ESI-Orbitrap-MS/MS method. Furthermore, the in vitro antioxidant and antibacterial activities were determined. The putative analysis led to the identification of 62 compounds: 41 in positive and 21 in negative ionization mode. Predominant classes included flavonoids, organic acids, terpenes, fatty acids, sugars, and amino acids. The antiradical tests revealed the greatest antioxidant potential for the methanol extract, followed by the ethanol one, consistent with the findings for the total polyphenol and flavonoid contents. The antibacterial assays revealed the lowest minimum inhibitory concentration against the studied Gram-positive bacteria for the methanol extract, while the ethanol one better suppressed the Gram-negative ones’ growth. Additionally, the ethanol extract had the lowest minimum bactericidal concentration (MBC) against *S. aureus* while the hydroalcoholic extracts’s MBC was equal for the rest of the studied bacterial strains. The present findings provide additional insights into the phytochemical contents and in vitro biological activity of *Achillea clypeolata*, and serve as a basis for future investigations associated with its pharmacological application.

## 1. Introduction

Attentive research into floral species with biological and ethnopharmacological activities has continuously been on focus given the tendentious need for novel compounds with therapeutic potential [1].

Genus *Achillea* is one of the most polymorphic and vast representatives of the Asteraceae family, consisting of more than 120 species. The genus is characterized by its wide abundance across the globe with its members being distributed in Europe, America, Australia, and Asia [2,3]. Fifty-six species have been identified in the region of the Balkan Peninsula, 18 of which are endemic [4]. According to the latest taxonomic studies, 22 species of perennial herbs belonging to the genus are a part of the flora of the Republic of Bulgaria [5]. Members of genus *Achillea* have long been used in traditional medicine with some also finding application in the food and cosmetic industries [6].

*Achillea clypeolata* Sibth. & Sm., also known as yellow, or moonshine yarrow is a Balkan endemite native to several Balkan countries such as Turkey, Greece, Serbia, Romania, and Bulgaria [7]. It is a diploid species with characteristic yellow flowers and up to 60 cm in height, belonging to the *A. filipendulina* group [8,9]. A typical habitat of *A. clypeolata* is open fields with sporadic trees and shrubs, with the plant growing in shallow soils on limestone [10].

*A. clypeolata* is a prominent and commonly used plant in Bulgarian traditional medicine as its many folk names suggest. Its ethnopharmacological usage ranges from treatment of gastrointestinal atony, liver diseases and hemorrhoids, to amenorrhea, bleeding and wounds, and different inflammatory conditions [7,10]. These pharmacological activities could be ascribed to the high content of flavonoids, sesquiterpenes, diterpenes, and phenolic compounds [10].

Currently, detailed investigations into the phytochemistry as well as the biological activity of whole plant extracts from *A. clypeolata* are still very scarce. Most of the published data concern the essential oil composition [10,11], with the studies having employed a gas chromatography–mass spectrometry technique [10,12,13]. Our literature review discovered only two studies that have established the phenolic contents of a methanol extract [2] or a chloroform extract [9] of the plant through a liquid chromatography–mass spectrometry/mass spectrometry (LC-MS/MS) method. The investigation into *A. clypeolata*’s biological activity has centered on its antioxidant and antimicrobial properties [11,13,14,15], with some studies focusing on the plant’s anti-inflammatory [10] and dermatological activity [2].

In Bulgaria, the studies on *A. clypeolata* have been concentrated mainly on its taxonomy, morphological, and ecological characteristics. The used plant samples were collected from locations such as Pirin Mountain [16], Kaliakra cape, Balchik, Varna, Strandzha Mountain, and Slavyanka Mountain [17], Golo Burdo [9], Dobrostan and Ivaylovgrad [18]. The results of the studies demonstrate a clear chemical diversity within the species, manifested mainly in the different contents of the essential oil of the plant. The latter may be explained by different environmental conditions such as soil, altitude, precipitation, and temperature [10].

The deficiency of knowledge on the total phytochemical composition as well as the lack of comparative data on the biological activity of *A. clypeolata* extracts using different solvents justify the present study’s purpose. Furthermore, data on *A. clypeolata* naturally growing on the territory of Plovdiv district (the town of Asenovgrad and village of Markovo) are nonexistent. Consequently, the present study aimed to conduct a comprehensive Ultra-High-Performance Liquid Chromatography–Electrospray ionization (UHPLC-ESI)-Orbitrap-MS/MS untargeted metabolomic profiling and gain insight into the antioxidant and antibacterial activity of methanol, ethanol and water extracts of the Balkan endemic species *A. clypeolata* collected from its natural habitat in the district of Plovdiv.

## 2. Materials and Methods

### 2.1. Reagents and Chemicals

The chemicals utilized for the conduction of analysis were acquired from Sigma-Aldrich (Steinheim, Germany) and included 2,9-dimethyl-1,10-phenanthroline (neocuproine), 2,4,6-tri(2-pyridy)-s-triazine (TPTZ), 6-hydroxy-2,5,7,8-tetramethylchroman-2-carboxylic acid (Trolox), 2,2-diphenyl-1-picrylhydrazyl (DPPH), gallic acid, Folin–Ciocalteu’s phenol reagent, quercetin-3-rutinoside, AlAl_3_, FeCl_3_, CuCl_2_, acetic acid, formic acid (LC-MS grade; Honeywell Fluka, Seelze, Germany), acetonitrile (LC-MS grade; Chromasolv, Honeywell, Muskegon, MI, USA), methanol (LC-MS grade; Chromasolv, Honeywell, Muskegon, MI, USA), and ethanol. Deionized water (18 MΩ) was prepared on an Evoqua Ultrapure Water System (Barsbüttel, Germany).

### 2.2. Plant Material and Preparation of the Extracts

The aerial parts of *Achillea clypeolata* were harvested from the plant’s natural habitat in the territory of the town of Asenovgrad and Markovo village, municipality of Plovdiv, Bulgaria, during its flowering season in the summer of 2024. The plant material was subjected to drying in a shaded area at ambient temperature (22 ± 2 °C) for 10 days. The dried plant was subsequently milled on GRINDOMIX GM200 laboratory mill (GRINDOMIX GM200, RETSCH GmbH, Haan, Germany) to a powder consistency with an average particle size of less than 400 µm. Water, ethanol, and methanol extracts were obtained through triple 24 h maceration of 10 g of the plant material in the respective extragent—distilled water (H_2_O extract), 70% ethanol (EtOH extract), and 70% methanol (MeOH extract) in a ratio 1:10 *w/v*. The objectives for the choice of solvent concentration for the hydroalcoholic extracts were based on conducted preliminary studies, previous experience and literature preview. Two concentrations of ethanol were tested as potential extragents, namely 70% and 95%. According to the obtained results, the extraction yield for the 95% ethanol extract was approximately 30% lower than the 70% one. Furthermore, the 95% ethanol extract had lower abundance of bioactive compounds, and lower antioxidant activity. Hence 70% ethanol was selected as the solvent of choice. Our previous studies [19,20] as well as literature data on the extraction of other *Achillea* species [21,22,23,24] served as a basis for the selected concentration of methanol. The maceration was conducted at ambient temperature (22 ± 2 °C) and included continuous stirring and three 15 min cycles of ultrasonication per 24 h on an ultrasonic bath (Siel, Gabrovo, Bulgaria). At the end of every 24 h extraction period, the obtained extracts were centrifuged for 15 min at 6000 rpm on an MPW-352 R centrifuge (MPW Med. Instruments, Warsaw, Poland) and filtrated using Whatman No. 1 filter paper. The obtained supernatants from each extraction were afterwards combined and subjected to evaporation at 50 °C until complete dryness on a rotary evaporator (Heidolph, Schwabach, Germany). Following evaporation, the extraction yield for each extract was calculated using the equation:

Y = [(Weight of the dry extract (g)/Weight of the ground plant (g)) × 100]

where Y is the extraction yield, % (*w/w*).

**Figure 1:**
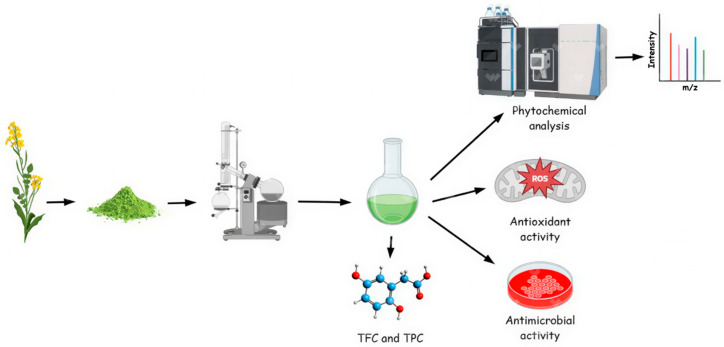
Experimental phases. TFC—total flavonoid content, TPC—total polyphenol content.

The three dry extracts were further lyophilized to dry powder on a Labconco freeze dryer (Labconco, Kansas City, MO, USA). The phases of the experiment are presented in Figure 1.

### 2.3. UHPLC-ESI-Orbitrap-MS/MS Phytochemical Analysis

The lyophilized extracts were dissolved in mobile phase (acetonitrile:water, 50:50 *v/v*) to a terminal concentration of 1 mg/mL. Chromatographic separation of 1 μL of the dissolved extracts was carried out on a HypersilGOLD reversed-phase column (100 × 2.1 mm, 1.9 μm particle size) maintained at 35 °C on a Vanquish Flex UHPLC system (Thermo Fisher Scientific, Waltham, MA, USA). The mobile phases contained 0.1% formic acid in water (A) and 0.1% formic acid in acetonitrile (B). The selected flow rate was 0.3 mL/min. The following gradient elution program was utilized: 0–3 min, 0–5% B; 3–25 min, 5–95% B; 25–30 min, 95% B; 30–31 min, 95–5% B. A 4 min re-equilibration step was included, resulting in a total run time of 35 min.

UHPLC-ESI-Orbitrap-MS/MS analyses were conducted on an Orbitrap Exploris 240 mass spectrometer (Thermo Fisher Scientific, Waltham, MA, USA) supplied with a heated electrospray ionization (HESI) source. Samples were analyzed in duplicates, in both positive (ESI+) and negative ionization modes (ESI-) within the m/z range from 80 to 1200.

The selected mass spectrometric parameters were: spray voltage—3500 V (+) and 2000 V (−); sheath gas flow rate—35 arbitrary units; auxiliary gas flow rate—10 arbitrary units; ion transfer tube temperature—320 °C; vaporizer temperature—350 °C, and S-lens RF level—70%.

Data were acquired in Full MS and data-dependent MS/MS (ddMS^2^) modes. Full MS scans were acquired at 120,000 resolution, while MS/MS scans were conducted at 30,000. The five most intense precursor ions per scan were adopted for MS/MS fragmentation (TopN = 5), with an intensity threshold of 5 × 10^5^ and normalized collision energies of 25%, 50%, and 75%. AcquireX 4.2 software was used to generate a background ion exclusion list from the matrix blank.

Data acquisition and processing were performed using Xcalibur 4.2 and Compound Discoverer 3.3 software (Thermo Fisher Scientific, Waltham, MA, USA). Compound identification was based on accurate mass measurement, MS/MS fragmentation patterns, and spectral library matching against mzCloud with minimum mzCloud best match factor threshold of 70%, ChemSpider, Predicted Compositions and mzVault databases. Annotation was performed using a Δ mass tolerance of ± 5 ppm, a minimum peak intensity threshold of 100,000, and a signal-to-noise (S/N) ratio threshold of 3. Furthermore, the Fragment Ion Search (FISh) option of the software was utilized for elucidation of the structure and structural transformation of the chromatographic peaks. The FISh coverage minimum threshold was set to ≥30.

### 2.4. Antioxidant Content and Antiradical Activity Assays

#### 2.4.1. Total Flavonoid and Total Polyphenol Content

The TFC was evaluated based on the method of Chang et al. [25] with AlCl_3_ reagent and quercetin dihydrate (2.5–200 µg/mL; R^2^ = 0.9993) as a standard. The results are expressed as mg/100 g dry weight (DW) quercetin equivalents (QEs).

The TPC was determined based on the method of Singleton and Rossi [26] with gallic acid (GA) as a standard and Folin–Ciocalteu reagent. The results are expressed as mg/100 g DW gallic acid equivalents (GAEs) (12.5–250 µg/mL; R^2^ = 0.9999).

#### 2.4.2. Ferric Reducing Antioxidant Power (FRAP)

FRAP was measured through Benzie and Strain’s method with FRAP reagent and Trolox (2.5–1000 µM/mL; R^2^ = 0.9999) as a standard [27] and as reported previously [19]. The results are expressed as µM/g DW Trolox equivalent (TE).

#### 2.4.3. Cupric Reducing Antioxidant Activity (CUPRAC)

CUPRAC was determined based on the method of Apak et al. with CuCl_2_ reagent, neocuproine, and Trolox (25–1000 µM/mL; R^2^ = 0.9929) as a standard [28]. The results are expressed as mM TE/g DW.

#### 2.4.4. 2,2-Diphenyl-1-picrylhydrazyl Radical Scavenging Activity (DPPH)

The DPPH radical scavenging activity (RSA) was measured following the method of Brand-Williams et al. with a DPPH reagent and control containing 1.7 mL 80 µM DPPH and 50 µL of 99% methanol [29]. The RSA was calculated with the following equation:

% RSA = [(Abs_control_ − Abs_sample_/Abs_control_) × 100]

The spectrophotometric analyses were conducteded on an Evolution 300 UV-Vis spectrophotometer (Thermo Fisher Scientific, Waltham, MA, USA). All analyses were conducted in triplicates, and the mean value is reported.

### 2.5. Evaluation of the Antibacterial Activity of the Plant Extracts

The antibacterial activity of the three *A. clypeolata* extracts was assessed via the agar diffusion method against two Gram-positive (*Staphylococcus aureus* ATCC55115 and *Streptococcus mutans* ATCC57853) and two Gram-negative (*Escherichia coli* ATCC56521 and *Pseudomonas aeruginosa* ATCC57853) bacterial strains (collection of the Department of Biochemistry and Microbiology, Faculty of Biology, University of Plovdiv “Paisii Hilendarski”). The bacteria were cultured on 5% sheep blood agar plates. The turbidity of the bacterial suspension was standardized to 0.5 McFarland. Bacterial inoculums were evenly spread over Mueller–Hinton (MH) agar plates to ensure uniform coverage. Wells with diameter of 10 mm were created in the agar through a sterile cork borer and each well was filled with 100 µL of the test solutions. The plates were kept at ambient temperature (22 ± 2 °C) for two hours before the 24 h incubation at 37 °C.

#### 2.5.1. Determination of the Minimum Inhibitory Concentration (MIC)

The MIC (lowest concentration of a substance that prevents visible microbial growth after 18 to 24 h of incubation) was assessed in sterile microplates via the microdilution method. The three extracts were first diluted in 2% dimethyl sulfoxide. In the first row of wells, 100 µL of each extract at a concentration of 50 mg/mL were added followed by 100 µL of MH broth. Serial dilutions were then performed. After incubation at 37 °C for 24 h, the optical density at 600 nm was measured.

#### 2.5.2. Determination of the Minimum Bactericidal Concentration (MBC)

The MBC, defined as the lowest concentration of an antibacterial agent that results in no more than 0.01% surviving bacteria, was measured following assessment of the MIC. Samples from each well showing no visible bacterial growth were transferred onto MH agar and incubated at 37 °C. After 24 h of incubation, the MBC was determined.

### 2.6. Statistical Analysis

The recorded peak intensities in the mass spectra of the *A. clypeolata* extracts were averaged across two independent replicates. The data was subjected to principal component analysis (PCA) as for overall assessment of data variability. For the visualization of differential metabolite abundance and hierarchical clustering patterns, the data was further subjected to heatmap analysis. Statistical comparisons concerning the biological activity tests were performed on SPSS statistical software (IBM, Chicago, IL, USA, version 17.0). The experiments were conducted in triplicates, and the data are presented as mean ± standard deviation (SD) values. The Shapiro–Wilk normality test was conducted for evaluation of data distribution. All *p* values were ≥ 0.05 which indicated Gaussian distribution. Based on the latter, statistical significance and intergroup comparisons for the biological contents and activity tests were evaluated with one-way ANOVA followed by Tukey’s post hoc test. Values for *p* ≤ 0.05 were considered statistically significant.

## 3. Results and Discussion

### 3.1. Extraction Yield and Total Bioactive Compounds

Table 1 demonstrates the findings for the extraction yield for the three chosen extraction solvents as well as the total content of polyphenols and flavonoids in the extracts. Under the same conditions of extraction such as temperature and time, the selection of a proper solvent is an important first step for optimal results to be obtained [30]. As evident from the data, the extraction yield was highest in the water extract followed by the methanol one. According to the obtained data, the water principally extracted small polar molecules. Among those were nitrogenous bases and nucleosides such as guanine, thymine and 2′-deoxyguanosine; sugars such as trehalose, ribulose, and mannose; amino acids, and organic acids such as quinic acid, lactic acid, malonic acid, and malic acid. On the other hand, extraction with both methanol and ethanol significantly increased the amount of both polyphenols and flavonoids. Therefore, 70% methanol and 70% ethanol used in our extraction method appeared to be better extragents than water (*p* ≤ 0.001). Furthermore, the comparison of the two hydroalcoholic extracts revealed higher TPC and TFC in the MeOH extract as opposed to the EtOH one (*p* ≤ 0.001).

It has been recognized that a substantial part of the biological activities of the *Achillea* genus is attributable to their high phenolic contents. Furthermore, flavonoids and phenolic acids appear to be among the most common secondary metabolites of the *Achillea* species [31]. According to the obtained data, extraction with 70% methanol resulted in the highest abundance of bioactive compounds. Additionally, our results show a higher TPC in the hydromethanolic extract compared to the results of a previous study that reported a phenolic content of 26.02 ± 1.04 mg GAE/g dry extract. However, the TFC registered in the same study was much higher than the one discovered in our study for the same type of extract (12.85 ± 0.69 mg QE/g dry extract) [2]. A likely reason for the discrepancy is the different geographical area from which the plant was collected. A similar chemical diversity for *A. clypeolata* species has been reported previously [10].

### 3.2. Phytochemical Analysis and Characterization of the Plant Extracts

#### 3.2.1. Total Ion Chromatograms (TICs) of the *A. clypeolata* Extracts

**Figure 2:**
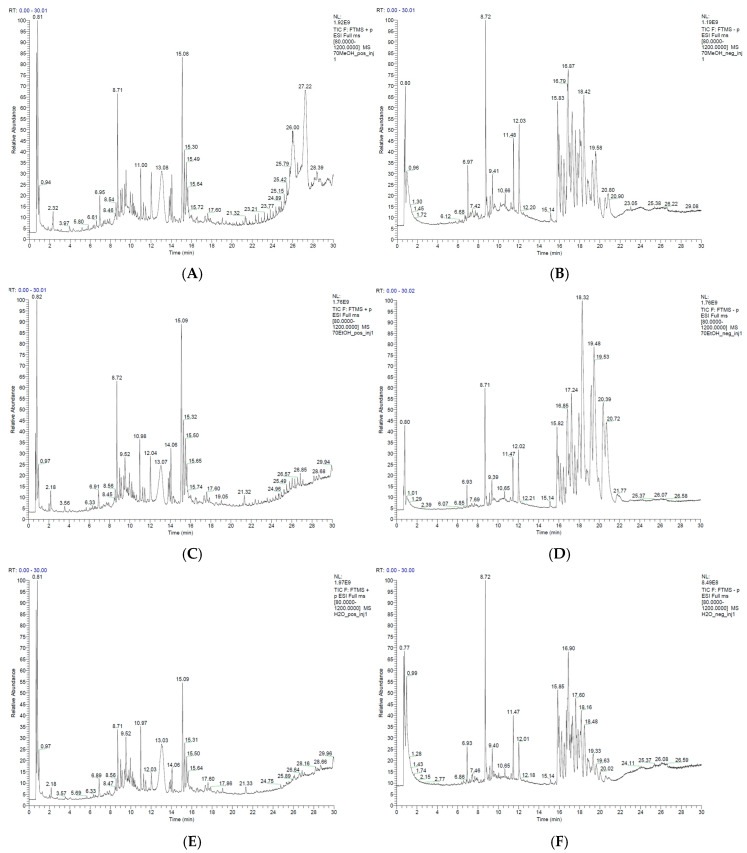
Total ion chromatograms of the *A. clypeolata* extracts: (**A**) MeOH in positive ionization mode; (**B**) MeOH in negative ionization mode; (**C**) EtOH in positive ionization mode; (**D**) EtOH in negative ionization mode; (**E**) H_2_O in positive ionization mode; (**F**) H_2_O in negative ionization mode.

The UHPLC-MS chromatograms of the methanol, ethanol, and water plant extracts are presented in Figure 2. As can be seen from the results, the methanol extract was characterized by the most complex chemical profile and highest chemical range, underlining the extraction efficacy. As opposed to the other two extracts, the TIC of the methanol one in positive ionization mode (Figure 2A) presented wide late signals of most likely more non-polar compounds. The TIC of the ethanol extract in ESI+ was similar to the methanol one; however, its average intensity was lower (Figure 2C). On the other hand, the TIC of the water extract in positive mode of ionization (Figure 2E) showed a significantly poorer profile with smaller peak intensities. Among the identified compounds with highest intensities extracted by the water were primary metabolites of low molecular weight including amino acids such as proline (RT—0.816), nucleobases and nucleosides such as thymine (RT—1.738) and 2′-deoxyguanosine (RT—1.296), and various natural products—NP-013538 (RT—1.795), NP-019722 (RT—1.250), and NP-021018 (7.431). The hydroalcoholic extracts were characterized by higher abundance of molecules eluted at the interval of 8–16 min. Among those compounds were flavonoids such as vitexin and quercetin 3-O-rhamnoside-7-O-glucoside; phenolic compounds such as yangonin, zingerone, and 4-methoxycinnamaldehyde; terpenes such as nootkatone and geranic acid, as well as aromatic compounds such as 4-phenylbutiric acid, 4-methylcinnamic acid, and acetophenone.

The total chromatogram of the hydroalcoholic extracts in ESI- (Figure 2B,D) was characterized by a cluster of well separated peaks in the 15–20 RT window, for which MS data indicate the presence of a homologous series of compounds. The primary compounds eluted at those RT included ions with m/z of 277.1844, 291.2001, 305.2158, and 319.2314, all of which showed a clear monoisotopic profile and differed by 14.0157 Da, corresponding to a methylene group. Furthermore, in all MS^2^ spectra, a characteristic fragment ion registered for all four compounds was 79.9574, compatible with a sulfonate group (SO_3_^−^). The lack of a more specific fragmentation pattern for those molecules, however, complicates their putative identification. Future analysis utilizing a higher collision energy will be necessary for fuller fragmentation and increased identification probability to be reached. The combination of accurate masses, a common fragment, and similar chromatographic features, however, supports the assumption that the peaks in this region originate from the same class of lipophilic compounds, probably sulfonate analogs with different hydrocarbon chain lengths.

The chromatogram of the water extract in ESI- (Figure 2F) was characterized by much less intensive peaks in the 15–20 min interval. From the identified compounds, prevailing in the MeOH extract were several flavonoids with RT from 8.5 to 9.2. Among those, most abundant was rutin (RT—8.72). In comparison, the other two extracts showed a lower distribution of this class of compounds. The water extract showed higher abundance of low-molecular-weight compounds eluted in the interval 0.7–1.2. Among those were mannose (RT—0.766) as well as several carboxylic acids including lactic acid (RT—0.783), malonic acid (RT—1.084), and malic acid (RT—1.208).

#### 3.2.2. Putative Metabolite Identification in the *A. clypeolata* Extracts

Table 2 presents the plant metabolites tentatively identified in the assayed *A. clypeolata* extracts. The employed untargeted molecular profiling resulted in the annotation of 62 compounds in total, of which 41 in ESI+ and 21 in ESI-. The compounds were putatively annotated on Compound Discoverer 3.3 software reaching identification level 2. Metabolite identification was based on the accurate mass of the compounds, their MS/MS fragmentation patterns, and spectral library matching against several online databases including mzCloud, ChemSpider, Predicted Compositions and mzVault.

The phytochemical screening of *A. clypeolata* led to the tentative identification of various classes of bioactive compounds, among which were flavonoids and flavonoid glycosides, phenolic acids, terpenes, coumarins, carboxylic acids, etc. These findings corroborate existing data on various other members of genus *Achillea*, for which the listed classes of compounds have been reported as dominant constituents [32]. Eight flavonoids and their glycosides were annotated in the present study. Among them rutin, vitexin, nicotiflorin, apigetrin and the quercetin and isorhamnetin glycosides have been previously identified as metabolites of different representatives of the *Achillea* genus [33,34,35,36].

Two phenolic acids were preliminary identified in the *A. clypeolata* extracts. The relative abundance calculated for chlorogenic acid (12.53%) makes it one of the most abundant metabolites in the studied extracts. Similarly, Barak et al. have reported that this quinic acid derivative was the major constituent in a methanol extract of the plant [2]. The annotated coumaric acid has been previously described as a fingerprint chemical in the *Achillea* species [33]. Other phenolic compounds annotated in the present study were yangonin, 4-methoxycinnamaldehyde, and zingerone.

Five terpenoid compounds with low relative abundance were tentatively identified in the studied plant extracts including nootkatone and geranic acid. To the best of our knowledge the former compound has not been previously annotated in representatives of the *Achillea* species, while geranic acid precursors have been reported to be present in some species of the genera [37]. In agreement with another study, the coumarin scopoletin was identified in the *A. clypeolata* extracts [9].

Several classes of primary metabolites were annotated in the present study including sugars and carbohydrate derivatives, amino acids and their derivatives, nucleobases and nucleosides, and fatty acids and lipid-like compounds. Among the identified sugars, α,α-trehalose, D-ribulose, 4-O-hexopyranosylhex-2-ulofuranose and D-(+)-mannose showed high relative abundance of 9.55, 9.43, 8.07, and 2.91% respectively. The amino acid proline was one of the most abundant metabolites in the studied extracts with calculated relative abundance of 23.38%. It has been reported that proline is a key metabolite in *Achillea* species, serving a protective role through osmotic balance maintenance, cellular pH regulation, reactive oxygen species scavenging and prevention of denaturation [38]. Furthermore, high proline contents could be associated with the reported wound-healing and dermatological activities of *A. clypeolata* and other yarrow species due to the amino acid’s role in collagen synthesis and tissue regeneration [2,31]. Pipecolic acid—a nonproteinogenic amino acid—was annotated in the *A. clypeolata* extracts at a relatively high abundance of 5.85%. Of the fatty acids and their derivatives, two metabolites appeared at relatively high abundance (26.56% and 2.18%). Based on the characteristic fragmentation pattern that was observed, in which a substantial number of the fragment ions differed by m/z of 14, we could deduce that those two annotated compounds could be classified as fatty acids or their derivatives. Confirmation will, however, be of nessecity. The oxylipin and jasmonic acid glucoside {(1R,2R)-2-[(2Z)-5-(Hexopyranosyloxy)-2-penten-1-yl]-3-oxocyclopentyl}acetic acid was also putatively identified as a component of the studied plant extracts at a relative abundance of 7.34%.

A derivative of cinnamic acid—4-methylcinnamic acid—was annotated in the present study at a relative abundance of 3.10%. A similar compound (2-hydroxycinnamic acid) has been previously identified to be present in an *A. clypeolata* extract [2], while cinnamic acid has been detected in other *Achillea* species [33]. Another tentatively identified aromatic compound was acetophenone, a compound formerly identified in *A. nobilis* [32].

O-succinylbenzoate was another compound annotated in the assayed plant extracts. Several benzoic acid derivatives, including 3- and 4-hydroxybeznoic acid, as well as 2,5-dihydroxybenzoic acid have been previously described in a methanol *A. clypeolata* extract [2].

The conducted analysis led to the tentative annotation of 8 carboxylic acids. Among them, quininc acid showed the highest relative abundance of 48.54%, followed by malic acid, at 13.26%. These data are consistent with the results reported by Yilmaz et al. [33]. Furthermore, salicylic acid has been previously identified as a component of several *Achillea* species [33,35]. Six unknown/database-coded natural products were also annotated; all were of relatively low abundance in the *A. clypeolata* extracts.

#### 3.2.3. Principal Component Analysis

**Figure 3:**
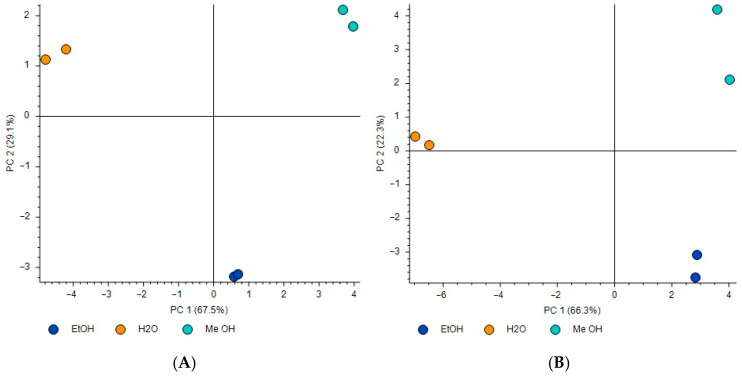
Unsupervised PCA of the *A. clypeolata* extracts in positive (**A**) and negative (**B**) ionization mode.

The employed unsupervised PCA performed on the 62 annotated plant metabolites revealed a distinct separation among the three different *A. clypeolata* extracts in both positive and negative ionization modes (Figure 3), which indicates a substantial chemical distinction. The first two principal components (PCs) had a total variance of 96.6% in positive ionization mode (Figure 3A), with the dominant chemical difference (PC1) evaluated as 67.5%, and the secondary variation (PC2) as 29.1%. With respect to the negative ionization mode (Figure 3B), the total PCA variance was 88.6%, of which PC1 accounted for 66.3% difference, while PC2 accounted for 22.3%.

#### 3.2.4. Heatmap Analysis

**Figure 4:**
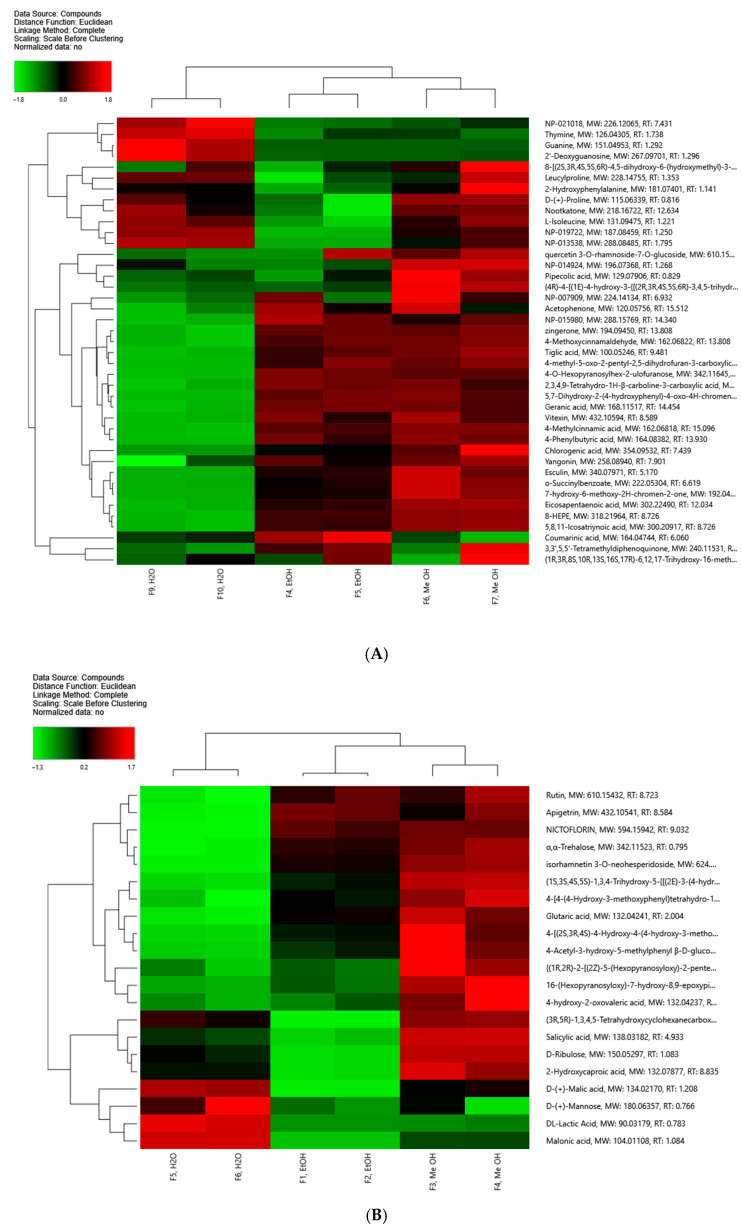
Clustered heatmap of the identified metabolites in the three *A. clypeolata* extracts in positive (**A**) and negative (**B**) ionization mode. Separate rows represent the metabolites, and the columns represent the extracts. Relative metabolite abundance is indicated by the different colors, where red shows higher levels, and green indicates lower levels of the respective compounds.

For the determination of the differential metabolites among the analyzed extracts, heatmap visualization with hierarchical clustering was employed as a part of the multivariate statistical analysis after the PCA. Euclidean distance was used for calculation of distance matrices, and Complete was used as the linkage method. Figure 4A presents the heatmap of the annotated compounds in ESI+, while Figure 4B shows those in ESI-. Each row of the map represents individual metabolites, and the columns correspond to the analyzed *A. clypeolata* extracts. Relative metabolite abundance is represented by the color gradients, with red representing higher levels, and green indicating lower levels of the respective compounds. As shown in Figure 4A,B, the heatmap analysis revealed distinct sample clustering, indicating pronounced metabolite profile distinction for the three plant extracts.

The conducted clustering showed pronounced sample grouping in accordance with the used extragent, with same extract type replicates clustering closely. Furthermore, the dendrogram demonstrated clear separation between the hydroalcoholic solvent extracts and the water one, which underlines the observed differences in metabolite profiles. Some of the annotated flavonoid glycosides and phenolic compounds such as apigetrin, isorhamnetin derivatives and rutin displayed higher relative abundance in the hydroalcoholic extracts in comparison to the water ones (Figure 4B). On the other hand, some low-molecular-weight sugars and organic acids, nitrogenous bases, dipeptides, and amino acids showed higher abundance in the aqueous *A. clypeolata* extracts (Figure 4A,B).

### 3.3. Antioxidant Capacity

**Figure 5:**
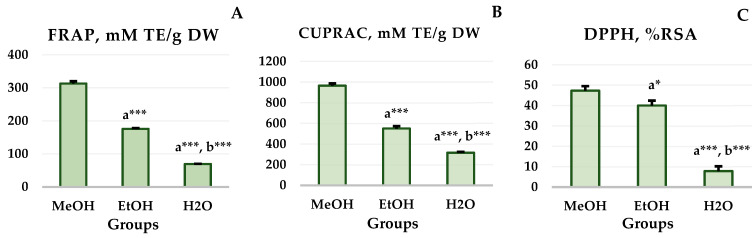
FRAP (**A**), CUPRAC (**B**), and DPPH (**C**) values of the *A. clypeolata* extracts. The data are presented as mean ± SD values. One-way ANOVA showed significant differences between groups for the FRAP values: F(2, 6) = 2463.981, *p* = 0.000, CUPRAC values: F(2, 6) = 859.734, *p* = 0.000, and DPPH values: F(2, 6) = 237.432, *p* = 0.000. The designation a* indicates significant differences versus the MeOH extract, *p* ≤ 0.05, a*** indicates significant differences versus the MeOH extract, *p* ≤ 0.001, and b*** indicates significant differences versus the EtOH extract, *p* ≤ 0.001 (one-way ANOVA, Tukey).

The results of the antioxidant capacity tests are presented in Figure 4. In agreement with the data on the TPC and TFC, the hydromethanolic plant extract exerted the most notable antiradical properties as compared to the other two extracts. According to the reducing power assays (FRAP and CUPRAC), the MeOH extract exhibited a significantly higher ferric ion reducing capacity than the EtOH extract (312.91 ± 7.03 vs. 175.90 ± 2.08, *p* ≤ 0.001) and the H_2_O extract (312.91 ± 7.03 vs. 69.34 ± 0.84, *p* ≤ 0.001; Figure 5A). Similarly, the hydroethanolic extract proved to possess a more significant reducing power than the water extract (175.90 ± 2.08 vs. 69.34 ± 0.84, *p* ≤ 0.001). Analogous results were obtained in the CUPRAC test (Figure 5B) with the methanolic extract showing highest reducing abilities compared to both the ethanolic (964.57 ± 21.86 vs. 549.77 ± 24.08, *p* ≤ 0.001) and the water extracts (964.57 ± 21.86 vs. 316.45 ± 8.38, *p* ≤ 0.001). The EtOH extract also proved to be a better electron donator than the water extract (549.77 ± 24.08 vs. 316.45 ± 8.38, *p* ≤ 0.001). The analysis of the DPPH radical scavenging activity (Figure 5C) revealed a significantly higher RSA for the methanolic extract in comparison to the ethanolic one (47.29 ± 2.22 vs. 39.96 ± 2.46, *p* ≤ 0.05) and the water one (47.29 ± 2.22 vs. 7.81 ± 2.40, *p* ≤ 0.001). The EtOH extract’s scavenging ability was significantly higher than that of the water one (39.96 ± 2.46 vs. 7.81 ± 2.40, *p* ≤ 0.001).

The data on the plant extracts’ antioxidant power further support the notion that 70% methanol was the most effective extragent. Our data corroborate the findings of Barak et al., which demonstrates that a methanol *A. clypeolata* extract exhibits both metal reducing and radical scavenging activities [2]. Higher DPPH and FRAP values of the methanol extract compared to the ethanol one registered in our study support the results of Menasri et al., which have also observed a more pronounced ferric reducing power and RSA of a methanol extract of another *Achillea* species [21]. Furthermore, similar FRAP values have been previously reported regarding an *Achillea santolina* methanolic extract [39]. Additionally, the CUPRAC study demonstrated a significantly higher antioxidant activity of the methanol extract compared to the other two. These data further support the cumulatively manifested antioxidant potential of *A. clypeolata* demonstrated in the study of Barak et al. who have also assessed the cupric reducing activity of the plant [2]. A remarkable antioxidant activity as evaluated in the CUPRAC assay has been reported for *Achillea monocephala* and *Achillea coarctata* [33].

A substantial body of reports link the antioxidant properties of *Achillea* species to the high abundance of phenolic compounds [2,6,40]. Several authors have reported on a strong positive correlation between the TPC and the antioxidant capacity of different *Achillea* species [6,41]. In this regard the higher antioxidant capacity of our methanol extract could be attributed to the higher phenolic contents compared to the other two *A. clypeolata* extracts. Furthermore, the lowest antioxidant activity as determined for the water extract could be explained by the fact that the most abundant compounds were primary metabolites such as nucleobases, sugars, amino acids and organic acids, which do not exhibit significant antioxidant properties.

### 3.4. Antibacterial Activity

The results of the antibacterial activity assays are presented in Table 3 and Table 4. As evident from the data, none of the *A. clypeolata* extracts exerted antibacterial activity against *E. coli*. The EtOH extract exhibited the highest MIC against the other studied Gram-positive bacterial strain (*P. aeruginosa*) in comparison to the MeOH and water extracts (*p* ≤ 0.001; Table 3). The latter two showed an equal inhibitory effect. Both hydroalcoholic extracts presented with the same MIC against *S. aureus* (*p* ≤ 0.001 vs. H_2_O; Table 3). The *A. clypeolata* methanol extract appeared to be most effective in suppressing the growth of *S. mutans* in comparison to both the ethanol (*p* ≤ 0.05) and the water extract (*p* ≤ 0.001 vs. H_2_O; Table 3).

The data on the MBC of the studied extracts are presented in Table 4. According to the results, both alcohol extracts had significantly lower MBC against *P. aeruginosa* than the water one (*p* ≤ 0.001). A significantly lower MBC against *S. aureus* was registered for the EtOH extract as compared to the MeOH and the water ones (*p* ≤ 0.001). Equal MBC values were observed for the hydroalcoholic extracts against *S. mutans*, which were significantly lower than the ones obtained for the water extract (*p* ≤ 0.01).

Many different pathogens are encountered by humans on a daily basis. The principal ones among them are the foodborne *S. aureus*, and *E. coli* [42], as well as *P. aeruginosa*, commonly found in water and soil [43], and *S. mutans* found in human oral microbiota and recognized as the primary cariogenic bacterium [44]. Interest in traditional medicine has been on the rise for the past decade and one of the reasons for this phenomenon is the observed changes in bacterial resistance, which calls for the need of new antimicrobial agents [15].

Our results corroborate the findings of Sotirova et al., according to which a methanol extract of *A. clypeolata* exerted a well-pronounced antibacterial activity against *P. aeruginosa*. At the same time, such activity was also detected against *E. coli* [13], contradictory to the results of the present work. Although not specified, the concentration of methanol used for the preparation of the extract in the latter study may be a contributing factor for the variation in results. Identical data were also reported by Simić et al., who observed antibacterial effect of *A. clypeolata* against *P. aeruginosa*, *E. coli*, and *S. aureus* [11]. However, the analysis was conducted with isolated essential oil, which is the likely reason for the discrepancy compared to our results. Lacking antibacterial activity of another *Achillea* species, namely *Achillea millefolium*, against *E. coli* has been previously reported by Mazandarani et al. [45].

To the best of our knowledge, this is the first study to report on the antibacterial activity of *A. clypeolata* extracts against *S. mutans*. Based on the data on the MIC and MBC, we could deduce that the methanol extract exhibited the most pronounced effect against the used bacterial strain. The antibacterial potential of other *Achillea* species against *S. mutans* has been researched before with extracts from *Achillea latiloba* [46], the essential oil of *Achillea ligustica* [47], and extracts from *Achillea filipendulina* [48] showing pronounced effects.

Several limitations of the present work have to be addressed. While the conducted study contributes to the available information on the chemical profile of *A. clypeolata*, the analysis only reached level 2 of identification. Hence, additional analysis using authentic standards for final metabolite identification would be the logical continuation of the present work. Furthermore, the observed biological activities only provide preliminary information that cannot be directly extrapolated to biological systems. The employed antioxidant assays were chemical-based and do not directly translate to in vivo redox conditions. Similarly, the observed antimicrobial activity may differ in the presence of in vivo mediated responses. Additionally, the lack of relevant standards in some of the assays like DPPH or the antimicrobial activity tests limits the interpretation of the obtained data.

## 4. Conclusions

The conducted untargeted phytochemical analysis of the methanol, ethanol and water *A. clypeolata* extracts led to the putative identification of 62 compounds, with 41 in positive and 21 in negative mode of ionization. Among them, chlorogenic acid, D-proline, D-quinic acid, and malic acid were characterized by high relative abundance, corroborating existing data on the *Achillea* species. Overall, the chemical profile of the assayed plant corresponds with the one reported for different representatives of the genera. Based on the findings on the biological activities, we can conclude that the methanol extract exerted the highest antioxidant potential, followed by the ethanol one, which correlates with the detected amount of total polyphenols and flavonoids. According to the obtained data, the hydroalcoholic extracts possess significant antibacterial activity against the studied bacterial strains with the exception of *E. coli*. The present study further supplements the existing information on the antibacterial activity of different *A. clypeolata* extracts by demonstrating a promising effect against *S. mutans*. These findings serve as a valuable basis for future investigations into the biological activity of the plant, including dermatological, antidiabetic and cytotoxic studies. Additional experiments will also be necessary for the definitive identification of the annotated compounds with authentic reference standards yet to be realized.

## Figures and Tables

**Table 1:** Extraction yield, total polyphenols and total flavonoids.

Species	Solvent	Yield, % (*w*/*w*)	TPC, mg GAE/g DW	TFC, mg QE/g DW
*Achillea* *clypeolata*	MeOH	24.6	34.27 ± 0.37 **^a^**	2.74 ± 0.04 **^a^**
	EtOH	15.9	19.38 ± 0.14 **^b^**	2.35 ± 0.04 **^b^**
	H_2_O	26.06	11.57 ± 0.86 **^c^**	1.61 ± 0.03 **^c^**

The data are presented as mean ± SD values. One-way ANOVA showed significant differences between groups for the TFC values: F(2, 6) = 653.257, *p* = 0.000, and the TPC values: F(2, 6) = 1347.757, *p* = 0.000. Different lower case superscript letters indicate significant differences between the extracts. The *p* values for all intergroup comparisons ≤ 0.001 (one-way ANOVA, Tukey).

**Table 2:** Bioactive compounds identified in the mass spectra of *Achillea clypeolata* extracts.

No.	Compound Name	Mode	RT [min]	Molecular Formula	Calculated MW	Annot. Δ Mass [ppm]	Fragmentation Pattern MS/MS	FISh Coverage	mzCloud Best Mach	Relative Abundance (%)
	**Flavonoids and flavonoid glycosides**									
1	8-[(2S,3R,4S,5S,6R)-4,5-dihydroxy-6-(hydroxymethyl) {[(2S,3R,4R,5R,6S)-3,4,5-trihydroxy-6-methyloxan-2- yl]oxy}oxan-2-yl]-5,7-dihydroxy-2-(4-hydroxyphenyl)-4H-chromen-4-one	(+)	7.459	C_27_H_30_O_14_	578.16156	−3.46	-	50.00	83.7	0.12
2	Apigetrin	(−)	8.584	C_21_H_20_O_10_	432.10541	−0.54	311.0565, 117.0347, 59.0138	41.18	73.6	0.12
3	Vitexin	(+)	8.589	C_21_H_20_O_10_	432.10594	0.69	313.0708, 283.0603, 165.0186	50.00	72.4	0.18
4	Quercetin 3-O-rhamnoside-7-O-glucoside	(+)	8.682	C_27_H_30_O_16_	610.15359	0.33	303.0502, 229.0500, 137.0237	39.02	82.6	3.07
5	Rutin	(−)	8.723	C_27_H_30_O_16_	610.15432	1.53	300.0278, 271.0250, 108.0217	36.73	88.0	2.10
6	Nicotiflorin	(−)	9.032	C_27_H_30_O_15_	594.15942	1.60	285.0408, 229.0508, 151.0035	36.00	88.5	0.09
7	5,7-Dihydroxy-2-(4hydroxyphenyl)-4-oxo-4H-chromen-3-yl 6-O-(6deoxyhexopyranosyl)hexopyranoside	(+)	9.044	C_27_H_30_O_15_	594.15884	0.63	287.0554, 121.0285, 85.0285	37.04	76.5	0.14
8	Isorhamnetin 3-O-neohesperidoside	(−)	9.172	C_28_H_32_O_16_	624.16994	1.45	315.0512, 271.0249, 151.0037	34.48	70.9	0.41
**Phenolic acids and simple phenolic compounds**										
9	Coumaric acid	(+)	6.060	C_9_H_8_O_3_	164.04744	0.55	137.0598, 119.0492, 91.0543	50.00	85.0	0.20
10	Chlorogenic acid	(+)	7.439	C_16_H_18_O_9_	354.09532	0.67	163.0390, 135.0441, 117.0335	59.52	96.8	12.53
11	Yangonin	(+)	7.901	C_15_H_14_O_4_	258.08940	0.75	199.0754, 171.0805, 145.0649	42.86	82.5	0.13
12	4-Methoxycinnamaldehyde	(+)	13.808	C_10_H_10_O_2_	162.06822	0.88	135.0805, 91.0543, 79.0543	40.00	78.5	0.43
13	Zingerone	(+)	13.808	C_11_H_14_O_3_	194.09450	1.05	163.0754, 135.0805, 105.0699	59.52	77.7	2.39
**Terpenes and terpenoid compounds**										
14	(1R,3R,8S,10R,13S,16S,17R)-6,12,17-Trihydroxy-16-methyl-8-(2-methyl-2-propanyl)-2,4,14,19-tetraoxahexacyclo[8.7.2.01,11 03,7 07,11 013,17]nonadecane-5,15,18-trione	(+)	6.515	C_20_H_24_O_10_	424.13494	−4.74	-	33.33	71.8	0.06
15	(4R)-4-[(1E)-4-hydroxy-3-{[(2R,3R,4S,5S,6R)-3,4,5-trihydroxy-6-(hydroxymethyl)oxan-2-yl]oxy}but-1-en-1-yl]-3,5,5-trimethylcyclohex-2-en-1-one	(+)	6.931	C_19_H_30_O_8_	386.19437	0.79	207.1380, 123.0804, 95.0857	66.67	70.8	0.21
16	16-(Hexopyranosyloxy)-7-hydroxy-8,9-epoxypimaran-18-oic acid	(−)	9.919	C_26_H_42_O_10_	514.27817	0.72	495.2601, 351.2170, 59.0138	50.00	92.2	0.03
17	Nootkatone	(+)	12.634	C_15_H_22_O	218.16722	0.71	161.1325, 121.1012, 93.0699	59.18	78.3	0.46
18	Geranic acid	(+)	14.454	C_10_H_16_O_2_	168.11517	0.81	137.0962, 123.0805, 95.0855	73.08	75.3	0.16
**Coumarins**										
19	Esculin	(+)	5.170	C_15_H_16_O_9_	340.07971	0.81	179.0340, 123.0441, 97.0285	61.54	94.1	0.23
20	7-Hydroxy-6-methoxy-2H-chromen-2-one (Scopoletin)	(+)	6.236	C_10_H_8_O_4_	192.04236	0.54	178.0261, 133.0285, 77.0386	40.85	77.6	0.30
**Alkaloids**										
21	2,3,4,9-Tetrahydro-1H-β-carboline-3-carboxylic acid	(+)	6.123	C_12_H_12_N_2_O_2_	216.09000	0.58	173.1075, 144.0809, 74.0237	52.63	91.7	0.18
**Sugars and carbohydrate derivatives**										
22	D-(+)-Mannose	(−)	0.766	C_6_H_12_O_6_	180.06357	0.99	161.0457, 89.0245, 59.0139	59.38	77.8	2.91
23	4-O-Hexopyranosylhex-2-ulofuranose	(+)	0.786	C_12_H_22_O_11_	342.11645	0.70	145.0498, 127.0391, 85.0285	68.97	92.2	8.07
24	α,α-Trehalose	(−)	0.795	C_12_H_22_O_11_	342.11523	−2.87	179.0563, 89.0244, 59.0138	80.00	91.3	9.55
25	D-Ribulose	(−)	1.083	C_5_H_10_O_5_	150.05297	0.96	131.0351, 89.0244, 71.0139	67.50	78.3	9.43
26	4-Acetyl-3-hydroxy-5-methylphenyl β-D-glucopyranoside	(−)	5.341	C_15_H_20_O_8_	328.11623	1.27	165.0558, 101.0246, 59.0138	50.00	78.5	0.04
27	4-[(2S,3R,4S)-4-Hydroxy-4-(4-hydroxy-3-methoxybenzyl)-3-(hydroxymethyl)tetrahydro-2-furanyl]-2-methoxyphenyl β-D-glucopyranoside	(−)	7.615	C_26_H_34_O_12_	538.20572	1.29	375.1449, 113.0245, 71.0139	30.65	72.2	0.13
28	4-[4-(4-Hydroxy-3-methoxyphenyl)tetrahydro-1H,3H-furo[3,4-c]furan-1-yl]-2-methoxyphenyl hexopyranoside	(−)	9.016	C_26_H_32_O_11_	520.19502	1.08	357.1335, 151.0402, 136.0167	56.76	88.8	0.12
**Amino acids and derivatives**										
29	D-(+)-Proline	(+)	0.816	C_5_H_9_NO_2_	115.06339	0.54	98.0603, 70.0652	85.71	89.7	23.38
30	Pipecolic acid	(+)	0.829	C_6_H_11_NO_2_	129.07906	0.63	84.0809	37.50	87.2	5.85
31	2-Hydroxyphenylalanine	(+)	1.141	C_9_H_11_NO_3_	181.07401	0.64	165.0549, 136.0759, 91.0543	42.50	89.0	0.21
32	L-Isoleucine	(+)	1.221	C_6_H_13_NO_2_	131.09475	0.91	114.0914, 86.0965, 69.0699	67.74	89.7	0.21
33	Leucylproline	(+)	1.353	C_11_H_20_N_2_O_3_	228.14755	0.68	116.0706, 70.0652	78.95	75.0	0.70
**Nucleobases and nucleosides**										
34	Guanine	(+)	1.292	C_5_H_5_N_5_O	151.04953	0.78	135.0303, 110.0350, 82.0401	47.62	90.9	0.49
35	2′-Deoxyguanosine	(+)	1.296	C_10_H_13_N_5_O_4_	267.09701	0.94	152.0568, 99.0442, 71.0492	50.00	88.0	0.17
36	Thymine	(+)	1.738	C_5_H_6_N_2_O_2_	126.04305	0.99	110.0238, 84.0445, 56.0496	35.48	94.0	0.14
**Fatty acids and lipid-like compounds**										
37	-	(+)	8.726	C_20_H_30_O_3_	318.21964	0.45	157.1013, 171.1169, 185.1326, 199.1483	-	91.0	26.56
38	5,8,11-Icosatriynoic acid	(+)	8.726	C_20_H_28_O_2_	300.20917	0.79	255.2110, 187.1123, 95.0855	49.44	86.3	2.60
39	Tiglic acid	(+)	9.481	C_5_H_8_O_2_	100.05246	0.33	83.0491, 55.0542	45.45	70.1	0.67
40	-	(+)	12.034	C_20_H_30_O_2_	302.22490	1.06	95.0856, 109.1012, 123.1168, 137.1328	-	88.7	2.18
41	{(1R,2R)-2-[(2Z)-5-(Hexopyranosyloxy)-2-penten-1-yl]-3-oxocyclopentyl}acetic acid	(−)	6.937	C18H28O9	388.17242	−2.34	143.0349, 113.0245, 101.0244	68.42	93.3	7.34
**Other aromatic and volatile compounds**										
42	(1S,3S,4S,5S)-1,3,4-Trihydroxy-5-{[(2E)-3-(4-hydroxy-3-methoxyphenyl)-2-propenoyl]oxy}cyclohexanecarboxylic acid	(−)	6.641	C_17_H_20_O_9_	368.11003	−1.90	193.0507, 134.0374, 117.0341	47.62	87.1	1.17
43	4-Phenylbutyric acid	(+)	13.930	C_10_H_12_O_2_	164.08382	0.54	137.0962, 109.1012, 93.0699	56.82	89.7	0.28
44	4-Methylcinnamic acid	(+)	15.096	C_10_H_10_O_2_	162.06818	0.65	121.0648, 91.0543, 79.0543	60.00	82.7	3.10
45	Acetophenone	(+)	15.512	C_8_H_8_O	120.05756	0.35	93.0699, 77.0386, 65.0385	73.68	85.6	0.30
**Miscellaneous**										
46	o-Succinylbenzoate	(+)	6.619	C_11_H_10_O_5_	222.05304	0.99	190.0263, 162.0313, 107.0492	64.71	83.9	0.39
47	4-methyl-5-oxo-2-pentyl-2,5-dihydrofuran-3-carboxylic acid	(+)	14.276	C_11_H_16_O_4_	212.10507	0.97	153.0911, 121.0649, 95.0493	44.44	71.6	0.14
48	3,3′,5,5′-Tetramethyldiphenoquinone	(+)	17.589	C_16_H_16_O_2_	240.11531	1.18	213.1275, 143.0856, 91.0542	41.86	80.0	1.32
**Carboxylic Acids**										
49	D/L-Lactic Acid	(−)	0.783	C_3_H_6_O_3_	90.03179	1.04	71.0139, 59.0139	83.33	79.2	1.01
50	D-(-)-Quinic acid	(−)	0.991	C_7_H_12_O_6_	192.06347	0.43	173.0457, 87.0088, 59.0138	33.33	92.0	48.54
51	Malonic acid	(−)	1.084	C_3_H_4_O_4_	104.01108	1.17	59.0139, 41.0033	50.00	89.1	0.44
52	D-(+)-Malic acid	(−)	1.208	C_4_H_6_O_5_	134.02170	1.32	115.0039, 89.0245, 71.0139	66.67	91.5	13.26
53	4-hydroxy-2-oxovaleric acid	(−)	1.531	C_5_H_8_O_4_	132.04237	0.84	113.0245, 87.0452, 69.0346	43.75	89.0	0.08
54	Glutaric acid	(−)	2.004	C_5_H_8_O_4_	132.04241	1.12	87.0452, 69.0346, 59.0139	43.75	87.0	0.27
55	Salicylic acid	(−)	4.933	C_7_H_6_O_3_	138.03182	0.89	93.0346	35.00	73.9	0.07
56	2-Hydroxycaproic acid	(−)	8.835	C_6_H_12_O_3_	132.07877	0.94	129.0559, 113.0610, 85.0659	70.00	87.5	2.92
**Unknown/Database-Coded Natural Products**										
57	NP-019722	(+)	1.250	C_8_H_13_NO_4_	187.08459	0.72	142.0864, 128.0707, 100.0758	87.50	75.8	0.44
58	NP-014924	(+)	1.268	C_10_H_12_O_4_	196.07368	0.63	165.0548, 137.0599, 109.0649	31.11	77.5	0.19
59	NP-013538	(+)	1.795	C_12_H_16_O_8_	288.08485	1.17	127.0391, 85.0285, 61.0285	56.25	84.3	0.05
60	NP-007909	(+)	6.932	C_13_H_20_O_3_	224.14134	0.43	207.1381, 123.0805, 95.0856	46.15	86.0	0.11
61	NP-021018	(+)	7.431	C12H18O4	226.12065	0.64	209.1175, 149.0963, 85.0649	75.00	75.6	0.37
62	NP-015980	(+)	14.340	C14H24O6	288.15769	1.39	123.0804, 109.1012, 69.0699	100.00	78.7	0.75

RT—retention time, MW—molecular weight, FISh—Fragment Ion Search.

**Table 3:** Minimum inhibitory concentration (mg/mL) of the *A. clypeolata* extracts.

Type of the Extract	Gram-Positive Bacteria		Gram-Negative Bacteria	
	*E. coli*	*P. aeruginosa*	*S. aureus*	*S. mutans*
*A. clypeolata* methanol extract	-	6.25 ± 0.00 ^b^	1.56 ± 0.05 ^a^	12.5 ± 0.00 ^a^
*A. clypeolata* ethanol extract	-	1.56 ± 0.00 ^a^	1.56 ± 0.01 ^a^	25.0 ± 0.00 ^b*^
*A. clypeolata* water extract	-	6.25 ± 0.05 ^b^	12.5 ± 0.00 ^b^	50.0 ± 0.00 ^c^

The data are presented as mean ± SD values. One-way ANOVA showed significant differences between groups for the MIC against *P. aeruginosa*: F(2,6) = 7673.058, *p* = 0.000, *S. aureus*: F(2,6) = 1395.998, *p* = 0.000, and *S. mutans*: F(2,6) = 65.299, *p* = 0.000. Different lower case superscript letters indicate significant differences between the extracts. The symbol * indicates a significant difference versus the methanol *A. clypeolata* extract, *p* ≤ 0.05. All other *p* values ≤ 0.001 (one-way ANOVA, Tukey).

**Table 4:** Minimum bactericidal concentration (mg/mL) of the *A. clypeolata* extracts.

Type of the Extract	Gram-Positive Bacteria		Gram-Negative Bacteria	
	*E. coli*	*P. aeruginosa*	*S. aureus*	*S. mutans*
*A. clypeolata* methanol extract	-	1.56 ± 0.05 ^a^	1.56 ± 0.01 ^b^	12.5 ± 0.00 ^a^
*A. clypeolata* ethanol extract	-	1.56 ± 0.00 ^a^	0.78 ± 0.05 ^a^	12.5 ± 0.00 ^a^
*A. clypeolata* water extract	-	6.25 ± 0.00 ^b^	6.25 ± 0.00 ^c^	25.0 ± 0.00 ^b**,##^

The data are presented as mean ± SD values. One-way ANOVA showed significant differences between groups for the MBC against *P. aeruginosa*: F(2, 6) = 6802.918, *p* = 0.000, *S. aureus*: F(2, 6) = 6303.048, *p* = 0.000, *and S. mutans*: F(2, 6) = 18.382, *p* = 0.003. Different lower case superscript letters indicate significant differences between the extracts. The symbol ** indicates significant differences versus the methanol *A. clypeolata* extract, *p* ≤ 0.01, and ## indicates significant differences versus the ethanol extract, *p* ≤ 0.01. All other *p* values ≤ 0.001 (one-way ANOVA, Tukey).

## Data Availability

The original contributions presented in this study are included in the article. Further inquiries can be directed to the corresponding author.

## Abbreviations

The following abbreviations are used in this manuscript:

LC-MS/MS	Liquid chromatography–mass spectrometry/mass spectrometry
UHPLC-ESI	Ultra-High-Performance Liquid Chromatography–Electrospray ionization
TPTZ	2,4,6-tri(2-pyridy)-s-triazine
DPPH	2,2-diphenyl-1-picrylhydrazyl
TFC	Total flavonoid content
TPC	Total polyphenol content
HESI	Heated electrospray ionization
ddMS^2^	Data-dependent MS/MS
S/N	Signal/noise
FISh	Fragment Ion Search
DW	Dry weight
QE	Quercetin equivalents
GA	Gallic acid
GAE	Gallic acid equivalents
FRAP	Ferric Reducing Antioxidant Power
TE	Trolox equivalents
CUPRAC	Cupric Reducing Antioxidant Activity
RSA	Radical scavenging activity
MH	Mueller–Hinton
MIC	Minimum Inhibitory Concentration
MBC	Minimum Bactericidal Concentration
PCA	Principal component analysis
SD	Standard deviation
TIC	Total ion chromatogram
PC	Principal component
RT	Retention time
MW	Molecular weight
